# Clinical and Functional Characteristics of Subjects with Asthma, COPD, and Asthma-COPD Overlap: A Multicentre Study in Vietnam

**DOI:** 10.1155/2018/1732946

**Published:** 2018-04-01

**Authors:** Sy Duong-Quy, Huong Tran Van, Anh Vo Thi Kim, Quyen Pham Huy, Timothy J. Craig

**Affiliations:** ^1^Bio-Medical Research Center, Lam Dong Medical College, Dalat, Vietnam; ^2^Division of Pulmonary, Allergy and Critical Care Medicine, Penn State College of Medicine, Hershey, PA, USA; ^3^Department of Health Science, Thang Long University, Hanoi, Vietnam; ^4^Nam Anh General Hospital, Binh Duong Province, Vietnam; ^5^Department of Clinical Immuno-Allergology, Hai Phong University, Haiphong, Vietnam

## Abstract

**Introduction:**

Subjects with asthma-chronic obstructive pulmonary disease (COPD) overlap (ACO) share common features of patients with asthma and COPD. Our study was planned to describe the clinical and functional features of subjects with ACO compared to asthma and COPD patients.

**Subjects and Methods:**

Study subjects who met the inclusion criteria were classified into three different groups: asthma, COPD, and ACO groups. All study subjects underwent clinical examination and biological and functional testing. They were then followed for 6 months to evaluate the response to conventional treatment.

**Results:**

From March 2015 to March 2017, 76 asthmatic (mean age: 41 ± 22 years), 74 COPD (59 ± 13 years), and 59 ACO (52 ± 14 years) subjects were included. The percentage of subjects with dyspnea on excretion in the ACO group was higher than that in asthma and COPD groups (*P* < 0.001 and *P* < 0.05, resp.). Subjects with COPD and ACO had significant airflow limitation (FEV_1_) compared to asthma (64 ± 17% and 54 ± 14% versus 80 ± 22%; *P* < 0.01 and *P* < 0.01, resp.). The levels of FENO in subjects with asthma and ACO were significantly higher than those in subjects with COPD (46 ± 28 ppb and 34 ± 12 ppb versus 15 ± 8 ppb; *P* < 0.001 and *P* < 0.001, resp.). VO_2_ max and 6MWD were improved in study subjects after 6 months of treatment. Increased CANO and AHI > 15/hour had a significant probability of risk for ACO (OR = 33.2, *P* < 0.001, and OR = 3.4, *P* < 0.05, resp.).

**Conclusion:**

Subjects with ACO share the common clinical and functional characteristics of asthma and COPD but are more likely to have sleep apnea. The majority of patients with ACO have a favourable response to combined treatment.

## 1. Introduction

The prevalence of asthma and chronic obstructive pulmonary disease (COPD) increased worldwide during the last decades and is expected to continue to rise in the next few decades. Asthma and COPD are a major problem for public health in many countries, especially for those with low-income status [1–3]. Recently, research has stressed that some patients might have clinical features of both asthma and COPD (asthma-COPD overlap syndrome or ACOS), particularly adult smokers with high reversibility of airflow obstruction and bronchial or systemic eosinophilic inflammation [4, 5]. It has been suggested that ACOS includes subjects with several different forms of airway diseases (phenotypes) caused by different underlying mechanisms (endotypes). Thus, ACOS has been somewhat defined as the coexistence of the features of two different diseases (asthma and COPD) in the same individual [6, 7].

Currently, to avoid the misunderstanding that ACOS is a single disease (syndrome), the term “ACO” (asthma-COPD overlap) has been recommended in a joint GINA and GOLD document [2]. In this document, ACO has been characterized by a persistent airflow limitation associated with several characteristics of asthma and COPD [2]. This concept is not a definition but instead a description to help classify patients in clinical practice. Although some studies attempted to describe the phenotype of ACO, the diagnosis and treatment of these patients are still controversial. Moreover, almost all subjects with ACO were excluded from previous clinical trials to evaluate the efficacy of therapy, and for this reason, the best treatment for ACO patients has not been determined. In addition, compared to patients with asthma and COPD, subjects with ACO have more symptoms, higher rate of acute exacerbations, greater health care consumption, and lower quality of life, suggesting that there is a definite need for further research [8–10].

Due to the diversity of recent published data of ACO from Asian countries, there are many important issues in the diagnosis and treatment of this disease in Asian patients. Therefore, a study of the features of subjects with ACO in an Asian country, such as in Vietnam, seems to be critical. This study describes the clinical and functional characteristics and the therapeutic response of subjects with ACO compared to those with asthma and COPD in a Vietnamese population.

## 2. Subjects and Methods

### 2.1. Subjects

Subjects more than 18 years old preselected from different centres in Vietnam who came to the Clinical Research Center of Lam Dong Medical College (LMC), Vietnam, for diagnosis and treatment for chronic respiratory diseases were included in this study after signing an Institutional Review Board- (IRB-) approved consent and meeting the inclusion and exclusion criteria. The present study had been approved by the LMC Institutional Review Board.

#### 2.1.1. Exclusion Criteria

Study subjects having one of the following criteria were excluded from the study: severe acute or chronic cardiovascular diseases (myocardial infarction, decompensated heart failure, or uncontrolled high blood pressure); severe acute asthma or COPD exacerbations requiring management in the Intensive Care Unit; current treatment with systemic corticosteroids; and those unable to perform the functional or biological or laboratory testing necessary for the study. Patients lost to follow-up during the study period were also excluded.

#### 2.1.2. Inclusion Criteria

All adult subjects had chronic respiratory symptoms confirmed by a detailed medical history and exam and were divided into three groups based on their presentation.

**Figure 1:**
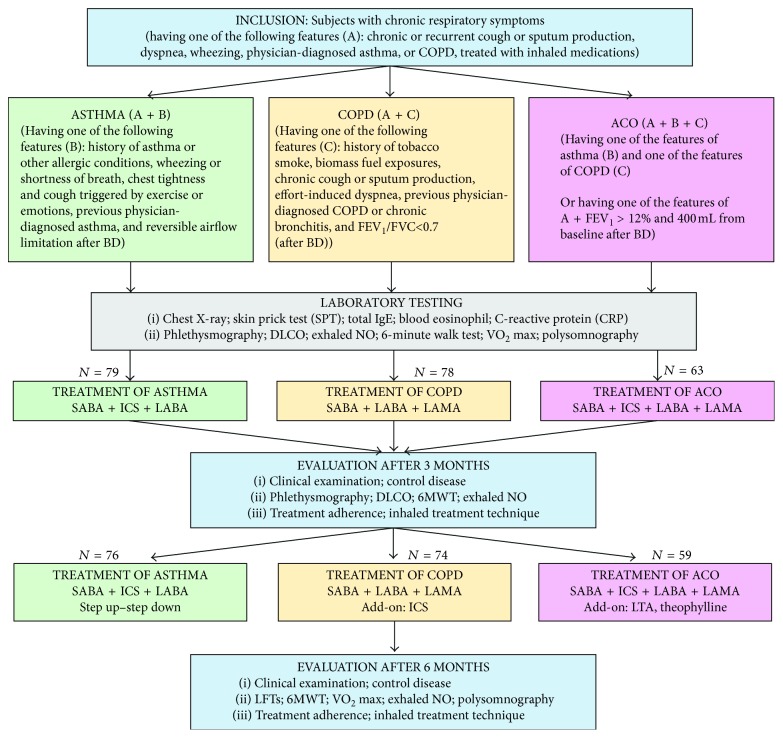
Flow chart for diagnosis and treatment of study subjects. COPD: chronic obstructive pulmonary disease; FEV_1_: forced expiratory volume in one second; FVC: forced vital capacity; BD: bronchodilator; DLCO: diffusing capacity of the lungs for carbon monoxide; 6MWT: 6-minute walking test; VO_2_ max: maximal oxygen consumption test; NO: nitric oxide; SABA: short-acting *β*_2_ agonist; LABA: long-acting *β*_2_ agonist; LAMA: long-acting muscarinic antagonist; ICS: inhaled corticosteroid; LFT: lung function test.

*Criteria A* required the following features: history of chronic or recurrent cough, sputum production, dyspnea, wheezing, report of a previous doctor diagnosis of asthma or COPD, history of treatment with inhaled medications, history of tobacco smoking, and occupational or domestic exposures to airborne pollutants (Figure 1).

*Criteria B* required the diagnosis of asthma based on GINA guidelines with one of the following features (criteria B; Figure 1): history of respiratory symptoms including wheeze, shortness of breath, chest tightness, and cough that varies over time and in intensity and reversibility of airway limitation defined as an increase of FEV_1_ >12% and 200 ml from baseline after bronchodilator (reversible airflow limitation) [11].

*Criteria C* is consistent with COPD and required a clinical diagnosis of COPD based on GOLD guidelines with one of the following features (criteria C; Figure 1) to include a history of dyspnea, chronic cough, and sputum production, a history of exposure to risk factors for the disease, and persistent airflow limitation with FEV_1_/FVC <0.70 after bronchodilator (BD) [12]:

*ACO*. Study subjects with chronic respiratory symptoms (criteria A) had been diagnosed as asthma-COPD overlap (ACO) if they had at least one of the asthma features (criteria B) associated with at least one of the COPD features (criteria C; Figure 1). ACO is also consistent with an increase of FEV_1_ >12% and 400 ml from baseline after BD (marked or high reversibility) in subjects who had chronic respiratory symptoms (criteria A) [11].

### 2.2. Methods

#### 2.2.1. Study Design

The study was cross-sectional, descriptive, and comparative. The study subjects were classified into an asthma group, COPD group, or ACO group according to the inclusion criteria (Figure 1). All data on family history, medical history, clinical examination, and laboratory tests were collected for statistical analyses.

#### 2.2.2. Laboratory Techniques

*(1) Biology and Skin Prick Test (SPT)*. Blood samples of all study subjects were collected through venipuncture and used for measuring total IgE and CRP and for counting eosinophils. The increases of total IgE, CRP, and eosinophils in peripheral blood were defined by a local biology lab (increased IgE: >214 KU/L; increased CRP: >10 mg/dL; and hypereosinophilia: >6%).

In the skin prick test (Stallergenes, UK), nine respiratory allergens including *Dermatophagoides pteronyssinus* (Dp), *Dermatophagoides farinae* (Df), *Blomia tropicalis* (Blo), dog hairs, cat hairs, cockroach, *Phoenix dactylifera*, Alternaria spp., and mixed pollens (*Dactylis glomerata*, *Phleum pratense*, and *Lolium perenne*) were performed for all study subjects. The skin prick test was considered positive when the wheal size exceeded the negative control by 3 mm. The negative control was 0.9% saline solution, and the positive control was 1 mg/ml of histamine.

*(2) Pulmonary Function Test and Exhaled Nitric Oxide (NO) Measurement*. Lung function testing was done by Body Box 500 (Medisoft, Sorinnes, Belgium) for whole-body phlethysmography. The reversibility of FEV_1_ (forced expiratory volume in one second) was evaluated after 15 min after BD with 400 *μ*g of salbutamol. The airflow limitation was considered reversible when the increase of FEV_1_ ≥12% and 200 mL (reversibility) or FEV_1_ ≥12% and 400 mL (marked or high reversibility). The measure of diffusing capacity of the lungs for carbon monoxide (DLCO) was performed as per the standard recommended guidelines of the American Thoracic Society (ATS)/European Respiratory Society (ERS) [13, 14].

Exhaled nitric oxide (NO) was measured with constant aspiratory flow using the HypAir FeNO^+^ Device (Medisoft), which is an electrochemical-based analyzer. The fraction of bronchial exhaled nitric oxide (FENO) and the concentration of alveolar nitric oxide (CANO) were measured with multiple flow rates. Technical measurement of exhaled NO was conducted according to the manufacturer's instructions and as recommended by the ATS/ERS guidelines [15].

*(3) Six-Minute Walk Test (6MWT) and Maximal Oxygen Consumption Test (VO_2_ Max)*. All study subjects performed the 6MWT and VO_2_ max tests at inclusion into the study and while in the stable status and after 3rd and 6th months of therapy. The 6MWT was accomplished as recommended by the ATS [16]. The 6-minute walk distance and the change of oxygen desaturation (DOD) were collected for analyses.

The VO_2_ max test was performed using an Ergo Card (Medisoft). It was based on the symptom-limited physical exercise test with ventilatory expired gas analysis using a cycle ergometer. The workload from 5 Watts to 10  Watts or 15 Watts/minute protocol had been adapted for each study subject to obtain at least 8 min (with 2 min of warming up for the first step) of exercise duration. Continuous standard 12-lead electrocardiograms, manual blood pressure measurements, and heart rate recordings were monitored at every stage. Data for oxygen consumption (VO_2_), carbon dioxide production (VCO_2_), minute ventilation (VE), respiratory rate (RR), and work load were collected continuously throughout the exercise. The peak of oxygen consumption uptake (VO_2_ max) was used for comparing the exercise capacity of study subjects.

(4) Sleep study with polysomnography (PSG). In-laboratory overnight PSG was performed for each study subject using Alice PSG (Philips, USA) as recommended [17]. The recorded and analyzed parameters included the apnea-hypopnea index (AHI), type of apnea (central apnea, obstructive apnea, or mixed apnea), oxygen saturation (SpO_2_) and minimum SpO_2_ (nadir SpO_2_), and sleep efficiency.

### 2.3. Statistical Analyses

All collected data were analyzed by SPSS 22.0 software (Chicago, USA). Categorical variables were expressed as numbers or percentages. Continuous variables were presented as mean ± SD. Normal distribution was evaluated by using the skewness-kurtosis test. The Mann–Whitney *U* test was used for pair comparison of mean between two groups, and the Kruskal–Wallis test was used for pair comparison of more than two groups. Binary logistic regression with a single categorical predictor was used to analyze the levels of probability risk factor for diseases (asthma, COPD, and ACO).

## 3. Results

### 3.1. Clinical Characteristics of the Study Subjects Classified by Group

From March 2015 to March 2017, 220 subjects were recruited in the present study, including 79 asthmatic subjects, 78 subjects with COPD, and 63 subjects with ACO (asthma-COPD overlap). After 6 months, there were 209 subjects (asthma: 76, COPD: 74, and ACO: 59) who completed the study and their data were analyzed, while 11 study subjects withdrew from the study and were lost to follow-up (Figure 1).

The mean age of asthmatic subjects was significantly lower than that of subjects with COPD and ACO (*P* < 0.01 and *P* < 0.01, resp.; Table 1). The male/female ratio was significantly greater in the COPD group compared with the asthma and ACO groups (8.4 versus 0.9 and 3.3; *P* < 0.001 and *P* < 0.05, resp.; Table 1). There was no significant difference between the three groups for BMI. The percentage of active smokers was greater in the COPD than that in the asthma or ACO groups (72.9% versus 5.3% and 37.2%, resp.; Table 1). As expected, the percentage of subjects having a medical history of chronic bronchitis was significantly higher in the COPD group than that in the two other groups (77.4% versus 17.1% and 47.4%; *P* < 0.001 and *P* < 0.01; Table 1), while the percentage of subjects with a medical history of asthma was significantly greater in the asthma cohort than that in either the COPD or ACO groups (Table 1). In COPD and ACO groups, the main respiratory symptoms were cough and sputum production, and dyspnea on excretion (90.5% and 84.7%; 82.4% and 91.5%; Table 1). The percentage of subjects with dyspnea on excretion in the ACO group was significantly higher than that in the COPD group (*P* < 0.05). However, the percentage of subjects having an exacerbation was greater in asthma and ACO groups than that in COPD subjects (89.4% and 57.6% versus 25.6%; *P* < 0.001 and *P* < 0.01, resp.; Table 1).

### 3.2. Biological and Functional Characteristics of the Subjects Classified by Group

The percentage of subjects with asthma having hypereosinophilia and increased total IgE was significantly higher than that in COPD and ACO subjects (78.9% versus 5.4% and 64.4%, *P* < 0.001 and *P* < 0.05; 81.5% versus 27.0% and 45.7%, *P* < 0.001 and *P* < 0.01, resp.; Table 2). The percentage of subjects having increased CRP was significantly higher in the COPD group than that in either the asthma or ACO groups (54% versus 13.1% and 23.7%; *P* < 0.001 and *P* < 0.01, resp.; Table 2). The percentage of positive SPT (positive with at least one allergen) in subjects with asthma and ACO was significantly higher than that in subjects with COPD (92.1% and 54.2% versus 6.7%; *P* < 0.001 and *P* < 0.001, resp.; Table 2). Positive SPT was also significantly greater in subjects with asthma than that in subjects with ACO (*P* < 0.001; Table 2).

The result of lung function testing (LFT) showed that the subjects with COPD and ACO had significantly more airflow limitation compared to subjects with asthma (FEV_1_: 64 ± 17% and 54 ± 14% versus 80 ± 22%; *P* < 0.01 and *P* < 0.01, resp.; MEF25-50: 44 ± 12% and 42 ± 11% versus 72 ± 7%; *P* < 0.01 and *P* < 0.01, resp.; Table 2). The levels of FENO in subjects with asthma and ACO were significantly higher than those in subjects with COPD (46 ± 28 ppb and 34 ± 12 ppb versus 15 ± 8 ppb; *P* < 0.001 and *P* < 0.001, resp.; Table 2). There was a significant increase of the alveolar concentration of NO (C_A_NO) in subjects with ACO compared to subjects with asthma and COPD (6 ± 3 ppb versus 4 ± 2 ppb and 3 ± 2 ppb; *P* < 0.05 and *P* < 0.05, resp.). The percentage of subjects having moderate or greater degree of obstructive sleep apnea (OSA), defined as AHI >15/hour, was significantly higher in subjects with ACO than that in subjects with asthma and COPD (64.4% versus 35.5% and 36.4%; *P* < 0.01 and *P* < 0.01, resp.; Table 2). In addition, the mean AHI in subjects with ACO was significantly higher than that in those with asthma and COPD (*P* < 0.05 and *P* < 0.05, resp.; Table 2), while the nadir SpO_2_ was significantly lower in the ACO group compared to the other two groups. The means of 6MWD and VO_2_ max in subjects with asthma were significantly higher than that those in subjects with COPD and ACO (6MWD: 388 ± 126 m versus 324 ± 144 m and 317 ± 155 m; *P* < 0.05 and *P* < 0.05, resp.; VO_2_ max: 64 ± 12% versus 52 ± 12% and 56 ± 14%; *P* < 0.05 and *P* < 0.05, resp.).

### 3.3. Evolution of Clinical and Functional Parameters of Study Subjects after Treatment

The results after 6 months of follow-up showed that all study subjects with asthma, COPD, and ACO had a significant lower percentage of clinical symptoms for cough and sputum production (15.7% versus 25.1%, *P* < 0.05; 54.1% versus 90.5%, *P* < 0.001; 52.6% versus 84.7%, *P* < 0.001, resp.; Table 3), exacerbations (19.7% versus 89.4%, *P* < 0.001; 10.8% versus 25.6%, *P* < 0.01; 37.2% versus 57.6%, *P* < 0.05, resp.; Table 3), and dyspnea on excretion (14.4% versus 35.5%, *P* < 0.01; 51.3% versus 82.4%, *P* < 0.001; 67.7% versus 91.5%, *P* < 0.01, resp.; Table 3).

The results of LFT demonstrated a significant improvement of MEF25-50 and DLCO in subjects with ACO (63 ± 7% versus 42 ± 11%, *P* < 0.01; 79 ± 13% versus 68 ± 14%, *P* < 0.05, resp.; Table 3). In subjects with asthma and ACO, there was a significant reduction of FENO after 6 months of treatment (18 ± 7 ppb versus 46 ± 28 ppb, *P* < 0.001; 15 ± 10 ppb versus 34 ± 12 ppb, *P* < 0.001, resp.; Table 3). Additionally, there was a significant reduction of CANO in subjects with ACO (3 ± 2 ppb versus 6 ± 3 ppb; *P* < 0.05; Table 3).

After 6 months of treatment, there was a significant improvement of VO_2_ max, 6MWD, and difference of oxygen desaturation (DOD) during exercise in subjects with COPD (67 ± 10% versus 52 ± 12%, *P* < 0.05; 389 ± 147 m versus 324 ± 144 m, *P* < 0.05; 4 ± 3% versus 7 ± 3%, *P* < 0.05, resp.; Table 3). There was a significant improvement of VO_2_ max and 6MWD in subjects with asthma (72 ± 8% versus 64 ± 12%, *P* < 0.05; 442 ± 103 m versus 388 ± 126 m, *P* < 0.05, resp.; Table 3) and only VO_2_ max in subjects with ACO (65 ± 15% versus 56 ± 14%; *P* < 0.05; Table 3). Subjects with ACO had improvement of percentage of AHI > 15, mean AHI, and nadir SpO_2_ after treatment (*P* < 0.01, *P* < 0.01, and *P* < 0.05, resp.; Table 3).

### 3.4. Probability of Clinical, Biological, and Functional Risk Factors for Asthma, COPD, and ACO

**Figure 2:**
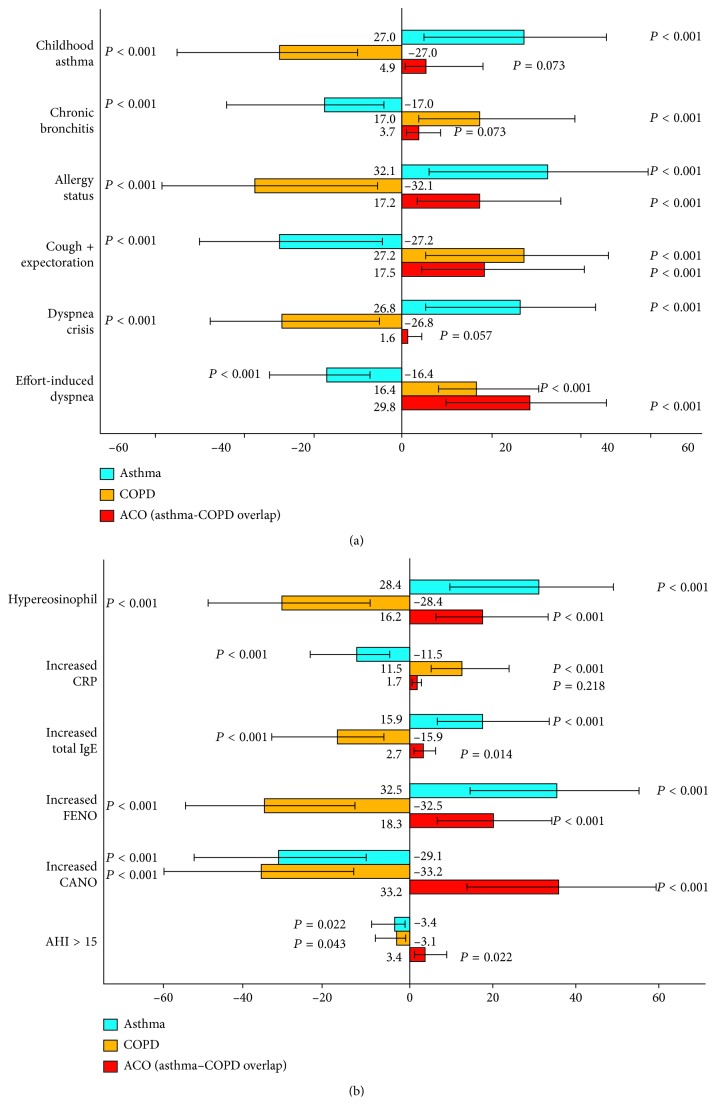
(a) Probability of clinical risk factors for asthma, COPD, and ACO. (b) Probability of functional risk factors for asthma, COPD, and ACO. F_E_NO: fractional exhaled NO; C_A_NO: alveolar concentration of NO; AHI: apnea-hypopnea index.

Clinical or biological features that defined COPD, such as previous diagnosed chronic bronchitis, symptoms of cough and sputum production, effort-induced dyspnea, and increased CRP, had a negative probability for risk factors of asthma (OR = −17.0, *P* < 0.001; OR = −27.2, *P* < 0.001; OR = −16.4, *P* < 0.001; OR = −11.5, *P* < 0.001, resp.; Table 4 and Figures 2(a) and 2(b)). The reverse was also true in that variables that suggested asthma, such as having a history of childhood asthma, atopic status, exacerbations, hypereosinophilia, increased total IgE, and high levels of F_E_NO, were a significant negative risk factor for COPD (OR = −27, *P* < 0.001; OR = −32, *P* < 0.001; OR = −26.8, *P* < 0.001; OR = −28.4, *P* < 0.001; OR = −15.9, *P* < 0.001; OR = −32.5, *P* < 0.001, resp.; Table 4 and Figures 2(a) and 2(b)). Compared to asthma and COPD, subjects with increased CANO and AHI >15/hour had a significant probability of having ACO (OR = 33.2, *P* < 0.001, and OR = 3.4, *P* < 0.05, resp.; Table 4 and Figure 2(b)).

## 4. Discussion

The results of our research demonstrated that almost all subjects with asthma and COPD had the main typical clinical features that lead to the appropriate diagnosis of the disease; however, a small number of subjects shared some of biological and functional features of both asthma and COPD. In the present study, the asthma group had only a small percentage of active smokers or former smokers, some of whom were previously diagnosed with COPD. In contrast, most subjects with asthma had a medical history of childhood asthma (Table 1). This result is similar with our previous studies in Vietnam [18, 19]. Most subjects in the asthma group were nonsmokers, with a medical history of childhood asthma, and had allergies, but some in the asthma cohort had adult-onset disease, were active or former smokers, and were not atopic as also noted in previous studies [20–23]. Moreover, in the present study, some asthmatic subjects were undiagnosed or misdiagnosed as having COPD with the end result of not being prescribed an ICS on a daily basis (44.7% of asthma patients) (Table 1). This problem has been especially concerning in low-resource countries such as in Vietnam [18]. A previous study reported that primary care doctors underdiagnosed asthma and failed to diagnose 25–35% of patients with asthma [24, 25]. Compared to COPD subjects, in the present study, the main symptom of subjects with asthma was dyspnea crisis, often referred to as an exacerbation, which is a typical characteristic of uncontrolled asthma [26].

The present study showed that subjects with COPD had the typical characteristics of this disease. They were older than asthmatic subjects and heavy active smokers and had a medical history of chronic bronchitis (Table 1). Similarly as with asthma patients, there was also a percentage of COPD subjects who were misdiagnosed as having asthma or undiagnosed, and this leads to the inappropriate use of ICS that could predispose to pneumonia. The result of the present study showed that when compared to subjects with asthma, the main symptom of subjects with COPD was chronic or recurrent cough associated with sputum production as noted in previous studies [3, 27, 28].

In contrast to asthma and COPD, study subjects with ACO, as diagnosed by the features recommended by GINA [2], shared the clinical characteristics of both diseases. Clinical characteristics of ACO were similar to those of asthma including a medical history of childhood asthma, exacerbations, and allergic status (Table 1). In turn, in our study, ACO has many features typical of COPD, and these include dyspnea on excretion, tobacco abuse, older age at diagnosis, and greater percentage of males. Until now, there are no clear clinical criteria to diagnose ACO. The initial definition of ACO proposed by the Spanish guideline in 2012 used the medical history of asthma as one of the major criteria and the history of atopy as one of the minor criteria of ACO [29]. In the recent Spanish guideline (GEMA 2015), the use of some clinical features such as symptoms before 40 years, previously diagnosed asthma, family history of asthma, and nocturnal symptoms in smokers or exsmokers has been proposed as criteria for diagnosis of ACO in combination with other functional and biological characteristics in the diagnosis algorithm [30]. The result of our study also shared some common clinical symptoms of the Spanish guideline. The GINA-GOLD approach to diagnoses of ACO suggested the use of certain clinical characteristics that might support the diagnosis of ACO including persistent or variability dyspnea on excretion, history of physician-diagnosed asthma, history of noxious exposures, and a significant reduction of symptoms from treatment, which are also noted in our Vietnamese patients [2, 3].

The result of the present study showed that the features of subjects with asthma were predominantly hypereosinophilia, increased total IgE, positive skin prick test to aeroallergens, reversibility of airflow obstruction, and high levels of F_E_NO (Table 2). These characteristics are typical biological and functional characteristics of asthma and used currently to categorize certain asthma phenotypes and also to tailor the target treatment, which in this case would be ICS. In contrast to the asthmatic subjects, in the present study, subjects with COPD were characterized mainly by increased CRP, irreversible airflow obstruction, and low levels of F_E_NO (Table 2). It is evident that multiple variables are required to assure the correct diagnosis and treatment. Inversely, the present study showed that subjects with ACO shared the similar biological characteristics of asthma such as hypereosinophilia, increased total IgE, and high levels of F_E_NO but also the functional characteristics of COPD such as distal airflow limitation, significant decreased DLCO, and low levels of 6MWD and VO_2_ max (Table 2). Interestingly, in comparison to asthma and COPD subjects, subjects with ACO had high levels of CANO and high percentage of moderate or greater obstructive sleep apnea (OSA) as diagnosed by the apnea-hypopnea index (AHI). Therefore, the high level of CANO may be a useful biomarker to help confirm the diagnosis in clinical practice.

The high level of F_E_NO in asthma has been known for more than 20 years and is used currently as a biomarker for diagnosis and treatment of asthma [31–34]. A high level of F_E_NO is a good biomarker for response to inhaled corticosteroids in asthmatic patients and is also suggested as a new biomarker recently approved for asthma [35]. Interestingly, CANO is not elevated in asthma as it is in ACO. High levels of CANO have also been demonstrated in interstitial pneumonia and OSA [36, 37]. In subjects with ACO, the increased level of CANO has not been described previously. We suggest that it might be due to a chronic inflammation or oxidative stress from distal airways. However, the precise mechanism of increased CANO in subjects with ACO should be clarified in the future by more studies. In contrast to other studies, in our cohort, only one subject with ACO (1.6%; data not shown) had a marked reversibility of FEV_1_, as defined as the increase of FEV_1_ >15% and 400 mL [38].

Although the result of the present study showed that all study subjects with asthma, COPD, or ACO had clinical and functional improvements after 6 months of treatment with different therapeutic options (dependent on diagnosis), a percentage of subjects with severe ACO was not significantly improved (Table 3). Moreover, there was no significant improvement of 6MWD during treatment in the ACO subjects. In a recent longitudinal study, Fu et al. [39] showed significantly a decline in 6MWD at four years in the COPD group compared with subjects with asthma and ACO, which conflicts with our data.

In the present study, the analysis of clinical, biological, and functional characteristics such as childhood asthma, allergy status, hypereosinophils, or high levels of FENO had a high probability of the diagnosis of asthma. Chronic bronchitis, chronic or recurrent sputum production, or effort-induced dyspnea had a high probability of a COPD diagnosis (Figures 2(a) and 2(b)), while high levels of FENO and CANO and their association with OSA (AHI >15/hour) had a high probability for a diagnosis of ACO. The present study suggests, for the first time, that high levels of CANO might be used as an additional biomarker for diagnosis of ACO. However, due to a small number of study subjects and lack of the current conventional or gold standard for diagnosis of ACO, the use of high levels of exhaled NO should not be used as a sole criterion to diagnose ACO. This is especially important since the sensitivity and specificity of CANO for the diagnosis of ACO require additional data.

## 5. Conclusion

ACO is a phenotype that shares the clinical, biological, and functional features of both COPD and asthma. Although the majority of patients with ACO have a favourable response to combined treatment, to include inhaled corticosteroids, some have a lack of adequate control of clinical symptoms. The high level of CANO may be a biomarker to identify patients with ACO. However, the target treatment of subjects with ACO and high level of CANO should be studied in the future.

## Figures and Tables

**Table 1:** Clinical characteristics of study subjects.

Parameters	Asthma (*N*=76)	COPD (*N*=74)	ACO (*N*=59)	*P*
Age (years)	41 ± 22	59 ± 13	52 ± 14	<0.01^∗,∗∗^; NS^∗∗∗^
Male/female ratio	0.9	8.4	3.3	<0.001^∗^; <0.05^∗∗,∗∗∗^
BMI (kg/m^2^)	22.4 ± 4.1	20.3 ± 3.5	21.7 ± 2.8	NS^∗,∗∗,∗∗∗^
*Smoking status*				
Never smoking (%)	76.3	8.1	38.9	<0.001^∗^; <0.01^∗∗,∗∗∗^
Active smoking (%)	5.3	72.9	37.2	<0.001^∗,∗∗^; <0.01^∗∗∗^
Former smokers (%)	18.4	19.0	23.9	NS^∗,∗∗,∗∗∗^
TC (pack-year)	9 ± 4	37 ± 12	31 ± 16	<0.001^∗,∗∗^; NS^∗∗∗^
*Diagnosis before inclusion*				
Undiagnosed	11.8	32.4	16.9	<0.05^∗,∗∗∗^; NS^∗∗^
Asthma (%)	75.1	24.3	52.5	<0.001^∗^; <0.05^∗∗^; <0.01^∗∗∗^
COPD (%)	13.1	43.3	32.6	<0.001^∗^; <0.05^∗∗,∗∗∗^
*Management before inclusion*				
Nontreated	44.7	56.7	33.8	NS^∗^; <0.05^∗∗,∗∗∗^
Treatment status	55.3	43.3	66.2	NS^∗^; <0.05^∗∗,∗∗∗^
SABA (%)	78.3	25.0	64.4	<0.001^∗,∗∗∗^; <0.05^∗∗^
LABA + LAMA (%)	15.6	75.0	28.8	<0.001^∗,∗∗∗^; <0.05^∗∗^
Others^*α*^ (%)	24.5	25.0	27.1	NS^∗,∗∗,∗∗∗^
*Medical history*				
Chronic bronchitis (%)	17.1	77.4	47.4	<0.001^∗^; <0.01^∗∗,∗∗∗^
Childhood asthma (%)	73.6	2.7	33.8	<0.001^∗^; <0.01^∗∗,∗∗∗^
Nonrespiratory diseases (%)	9.3	19.9	18.8	<0.01^∗,∗∗^; NS^∗∗∗^
Family history of asthma (%)	21.1	1.3	10.1	<0.001^∗^; <0.01^∗∗,∗∗∗^
Comorbidity	35.5	54.3	67.7	<0.05^∗,∗∗,∗∗∗^
CVD (%)	17.0	26.6	28.1	<0.01^∗,∗∗^; NS^∗∗∗^
ECD (%)	22.2	15.5	22.8	<0.05^∗,∗∗∗^; NS^∗∗^
Others (%)	40.8	39.9	19.9	NS^∗^; <0.05^∗∗,∗∗∗^
Allergy status (%)	82.8	12.1	77.9	<0.001^∗,∗∗∗^; <0.05^∗∗^
*Clinical symptoms*				
Cough + expectoration (%)	25.1	90.5	84.7	<0.001^∗,∗∗^; NS^∗∗∗^
Dyspnea crisis (exacerbation) (%)	89.4	25.6	57.6	<0.001^∗^; <0.01^∗∗,∗∗∗^
Dyspnea on excretion (%)	35.5	82.4	91.5	<0.001^∗,∗∗^; <0.05^∗∗∗^
HAE (times/year)	1.1 ± 0.7	1.2 ± 0.6	1.4 ± 1.3	NS^∗,∗∗,∗∗∗^

ACO: asthma-COPD overlap; BMI: body mass index; TC: tobacco consumption; SABA: short-acting beta-2 agonist; LABA: long-acting beta-2 agonist; LAMA: long-acting muscarinic antagonist; CVD: cardiovascular disease; ECD: endocrinology disorder defined as metabolic syndrome; HAE: hospitalization for acute exacerbation. ^*α*^Acetyl cysteine, theophylline, and leukotriene antagonists. ^∗^Asthma versus COPD; ^∗∗^asthma versus ACO; ^∗∗∗^ACO versus COPD.

**Table 2:** Biological and functional characteristics of study subjects.

Parameters	Asthma *N*=76	COPD *N*=74	ACO *N*=59	*P*
*Blood tests*				
Hypereosinophilia (%)	78.9	5.4	64.4	<0.001^∗,∗∗∗^; <0.05^∗∗^
Increased CRP (%)	13.1	54.0	23.7	<0.001^∗^; <0.01^∗∗∗^; <0.05^∗∗^
Increased total IgE (%)	81.5	27.0	45.7	<0.001^∗^; <0.01^∗∗,∗∗∗^
SPT (+) (%)	92.1	6.7	54.2	<0.001^∗,∗∗,∗∗∗^
*LFT after BD*				
FEV_1_	80 ± 22	64 ± 17	54 ± 14	<0.01^∗,∗∗^; <0.05^∗∗∗^
FEV_1_/FVC	72 ± 8	62 ± 6	64 ± 5	<0.05^∗,∗∗^; NS^∗∗∗^
MEF25-50	72 ± 7	44 ± 12	42 ± 11	<0.01^∗,∗∗^; NS^∗∗∗^
TLC	96 ± 14	118 ± 22	117 ± 16	NS^∗,∗∗,∗∗^
RV	126 ± 19	158 ± 23	158 ± 21	<0.05^∗,∗∗^; NS^∗∗∗^
FRC	142 ± 18	168 ± 21	167 ± 22	<0.05^∗,∗∗^; NS^∗∗∗^
Reversibility test (+) (%)	84	12	66	<0.001^∗,∗∗∗^; <0.05^∗∗^
DLCO	90 ± 12	73 ± 11	68 ± 14	<0.001^∗,∗∗^; NS^∗∗∗^
*Exhaled NO*				
Increased F_E_NO (%)	89.4	2.7	64.4	<0.001^∗,∗∗∗^; <0.05^∗∗^
Mean F_E_NO (ppb)	46 ± 28	15 ± 8	34 ± 12	<0.01^∗,∗∗∗^; <0.05^∗∗^
Increased C_A_NO (%)	6.5	4.1	83.0	NS^∗^; <0.001^∗∗,∗∗∗^
Mean C_A_NO (ppb)	4 ± 2	3 ± 2	6 ± 3	NS^∗^; <0.05^∗∗,∗∗∗^
*Exercise testing*				
VO_2_ max (%)	64 ± 12	52 ± 12	56 ± 14	<0.05^∗,∗∗^; NS^∗∗∗^
*6MWT*				
6MWD (metre)	388 ± 126	324 ± 144	317 ± 155	<0.05^∗,∗∗^; NS^∗∗∗^
DOD (%)	4 ± 2	7 ± 3	8 ± 4	<0.05^∗,∗∗^; NS^∗∗∗^
*Sleep study*				
AHI > 15 (%)	35.5	36.4	64.4	NS^∗^; <0.01^∗∗,∗∗∗^
AHI (times/hour)	16 ± 9	17 ± 8	24 ± 12	NS^∗^; <0.05^∗∗,∗∗∗^
Nadir SpO_2_ (%)	86 ± 9	84 ± 7	78 ± 8	NS^∗^; <0.05^∗∗,∗∗∗^

ACO: asthma-COPD overlap; SPT: skin prick test (positivity with ≥ one allergen); LFT: lung function test; BD: bronchodilator; FEV_1_: forced expiratory volume in one second; FVC: forced vital capacity; MEF: mean expiratory flow; TLC: total lung capacity; RV: residual volume; FRC: functional residual capacity; DLCO: diffusing capacity of the lungs for carbon monoxide; NO: nitric oxide; F_E_NO: fractional exhaled NO; C_A_NO: alveolar concentration of NO; 6MWD: 6-minute walk distance; DOD: differentiation of oxygen desaturation; AHI: apnea-hypopnea index. ^∗^Asthma versus COPD; ^∗∗^asthma versus ACO; ^∗∗∗^ACO versus COPD.

**Table 3:** Evolution of clinical and functional parameters after 6 months.

	Asthma (*N*=76)		*P*	COPD (*N*=74)		*P*	ACO (*N*=59)		*P*
	Inclusion	6th month		Inclusion	6th month		Inclusion	6th month	
*Clinical symptoms*									
Cough + expectoration (%)	25.1	15.7	<0.05	90.5	54.1	<0.001	84.7	52.6	<0.001
Dyspnea crisis (%)	89.4	19.7	<0.001	25.6	10.8	<0.01	57.6	37.2	<0.05
Effort-induced dyspnea (%)	35.5	14.4	<0.01	82.4	51.3	<0.001	91.5	67.7	<0.01
*Disease severity* ^¥^									
Severe asthma (%)	25.0	7.8	<0.001	—	—	—	—	—	—
Severe COPD (%)	—	—	—	24.3	11.5	<0.01	—	—	—
Severe ACO (%)	—	—	—	—	—	—	27.2	22.0	NS
*PFT after BD*									
FEV_1_	80 ± 22	89 ± 14	<0.05	64 ± 17	72 ± 18	<0.05	54 ± 14	69 ± 15	<0.01
FEV_1_/FVC	72 ± 8	74 ± 6	NS	62 ± 6	63 ± 7	NS	64 ± 5	66 ± 7	NS
MEF25-50	72 ± 7	74 ± 8	NS	44 ± 12	66 ± 8	<0.01	42 ± 11	63 ± 7	<0.01
TLC	96 ± 14	104 ± 12	NS	118 ± 22	112 ± 24	NS	117 ± 16	108 ± 17	NS
RV	126 ± 19	124 ± 20	NS	158 ± 23	149 ± 23	NS	158 ± 21	155 ± 22	NS
FRC	142 ± 18	140 ± 22	NS	168 ± 21	157 ± 22	NS	167 ± 22	159 ± 24	NS
DLCO	90 ± 12	89 ± 14	NS	73 ± 11	76 ± 13	NS	68 ± 14	79 ± 13	<0.05
*Exhaled NO*									
F_E_NO	46 ± 28	18 ± 7	<0.001	15 ± 8	14 ± 7	NS	34 ± 12	15 ± 10	<0.001
C_A_NO	4 ± 2	3 ± 2	NS	3 ± 2	4 ± 2	NS	6 ± 3	3 ± 2	<0.05
*Exercise testing*									
VO_2_ max	64 ± 12	72 ± 8	<0.05	52 ± 12	67 ± 10	<0.05	56 ± 14	65 ± 15	<0.05
6MWD	388 ± 126	442 ± 103	<0.05	324 ± 144	389 ± 147	<0.05	317 ± 155	332 ± 125	NS
DOD	4 ± 2	3 ± 3	NS	7 ± 3	4 ± 3	<0.05	8 ± 4	6 ± 5	NS
*Sleep study*									
AHI > 15 (%)	35.4	25.0	<0.05	36.5	28.9 ± 6	<0.05	52.7	37.2	<0.01
AHI (times/hour)	16 ± 9	17 ± 8	NS	17 ± 8	15 ± 7	<0.05	24 ± 12	16 ± 11	<0.01
Nadir SpO_2_ (%)	86 ± 9	88 ± 8	NS	85 ± 7	86 ± 8	NS	81 ± 8	87 ± 7	<0.05
*Treatment option*									
+SABA as needed	ICS_LD_ + LABA	ICS_MD_ + LABA + LTA	—	LABA + LAMA +	ICS_LD_ + LABA + LAMA + others^∗^	—	ICS_LD_ + LABA + LAMA	ICS_MD_-_HD_ LABA + LAMA + others^#^	—

ACO: asthma-COPD overlap; LFT: lung function test; BD: bronchodilator; FEV_1_: forced expiratory volume in one second; FVC: forced vital capacity; MEF: mean expiratory flow; TLC: total lung capacity; RV: residual volume; FRC: functional residual capacity; DLCO: diffusing capacity of the lungs for carbon monoxide; NO: nitric oxide; F_E_NO: fractional exhaled NO; C_A_NO: alveolar concentration of NO; 6MWD: 6-minute walk distance; DOD: differentiation of oxygen desaturation; AHI: apnea-hypopnea index; SABA: short-acting beta-2 agonist; LABA: long-acting beta-2 agonist; LAMA: long-acting muscarinic antagonist; ICS: inhaled corticosteroid; LD: low dose; MD: moderate dose; HD: high dose; LTA: leukotriene antagonist. ^¥^Disease severity was defined by LFT and based on FEV_1_ percent predicted after BD; severe asthma, COPD, and ACO were defined by FEV_1_ < 60%, <50%, and <50% predicted, respectively. ^#^Theophylline and acetyl cysteine. ^∗^Theophylline, acetyl cysteine, and macrolide.

**Table 4:** Probability of risk factors for asthma, COPD, and ACO.

Parameters	Odds ratio (CI 95% odds ratio), *P*		
	Asthma	COPD	ACO
Childhood asthma	27.0 (4.6–49.7) *P* < 0.001^∗^	−27.0 (4.6–49.7) *P* < 0.001^∗∗^	4.8 (0.9–17.2) *P*=0.073^∗∗∗^
Chronic bronchitis	−17.0 (3.5–37.9) *P* < 0.001^∗^	17.0 (3.5–37.9) *P* < 0.001^∗∗^	3.7 (1.0–8.2) *P*=0.058^∗∗∗^
Allergy status	32.1 (5.7–58.2) *P* < 0.001^∗^	−32.1 (5.7–58.2) *P* < 0.001^∗∗^	17.2 (3.5–35.4) *P* < 0.001^∗∗∗^
Cough + expectoration	−27.2 (4.5–51.9) *P* < 0.001^∗^	27.2 (4.5–51.9) *P* < 0.001^∗∗^	17.5 (3.8–40.3) *P* < 0.001^#^
Dyspnea crisis	26.8 (5.2–47.8) *P* < 0.001^∗^	−26.8 (5.2–47.8) *P* < 0.001^∗∗^	1.6 (0.5–3.7) *P*=0.057^∗∗∗^
Effort-induced dyspnea	−16.4 (7.2–30.7) *P* < 0.001^##^	16.4 (7.2–30.7) *P* < 0.001^∗∗^	29.8 (9.8–48.9) *P* < 0.001^##^
Hypereosinophilia	28.4 (9.4–48.6) *P* < 0.001^∗^	−28.4 (9.4–48.6) *P* < 0.001^∗∗^	16.2 (5.6–30.6) *P* < 0.001^∗∗∗^
Increased CRP	−11.5 (3.7–19.4) *P* < 0.001^∗^	11.5 (3.7–19.4) *P* < 0.001^∗∗^	1.7 (0.7–3.8) *P*=0.218^#^
Increased total IgE	15.9 (6.1–31.4) *P* < 0.001^∗^	−15.9 (6.1–31.4) *P* < 0.001^∗∗^	2.7 (1.2–5.9) *P*=0.014^∗∗∗^
Increased FENO	32.5 (12.6–50.5) *P* < 0.001^∗^	−32.5 (12.6–50.5) *P* < 0.001^∗∗^	18.3 (6.9–32.7) *P* < 0.001^∗∗∗^
Increased CANO	−29.1 (10.2–46.7) *P* < 0.001^##^	−33.2 (12.3–54.8) *P* < 0.001^###^	33.2 (12.3–54.8) *P* < 0.001^∗∗∗^
AHI > 15	−3.4 (1.3–9.9) *P*=0.022^##^	−3.1 (1.2–8.6) *P* < 0.05^###^	3.4 (1.3–9.9) *P*=0.022^#^

ACO: asthma-COPD overlap; ^∗^asthma versus COPD; ^∗∗^COPD versus asthma; ^∗∗∗^ACO versus COPD; ^#^ACO versus asthma; ^##^asthma versus ACO; ^###^COPD versus ACO.
